# Powering Research through Innovative Methods for Mixtures in Epidemiology (PRIME) Program: Novel and Expanded Statistical Methods

**DOI:** 10.3390/ijerph19031378

**Published:** 2022-01-26

**Authors:** Bonnie R. Joubert, Marianthi-Anna Kioumourtzoglou, Toccara Chamberlain, Hua Yun Chen, Chris Gennings, Mary E. Turyk, Marie Lynn Miranda, Thomas F. Webster, Katherine B. Ensor, David B. Dunson, Brent A. Coull

**Affiliations:** 1 Division of Extramural Research and Training, National Institute of Environmental Health Sciences, National Institutes of Health, Durham, NC 27709, USA; toccara.chamberlain@nih.gov; 2 Department of Environmental Health Sciences, Columbia University Mailman School of Public Health, New York, NY 10032, USA; mk3961@cumc.columbia.edu; 3 Division of Epidemiology and Biostatistics, School of Public Health, University of Illinois Chicago, Chicago, IL 60612, USA; hychen@uic.edu (H.Y.C.); mturyk1@uic.edu (M.E.T.); 4 Department of Environmental Medicine and Public Health, Icahn School of Medicine at Mount Sinai, New York, NY 10029, USA; chris.gennings@mssm.edu; 5 Department of Applied and Computational Mathematics and Statistics, University of Notre Dame, South Bend, IN 46556, USA; mlm@nd.edu; 6 Department of Environmental Health, Boston University School of Public Health, Boston, MA 02118, USA; twebster@bu.edu; 7 Department of Statistics, Rice University, Houston, TX 77005, USA; ensor@rice.edu; 8 Department of Statistical Science, Duke University, Durham, NC 27710, USA; dunson@stat.duke.edu; 9 Department of Biostatistics, Harvard T.H. Chan School of Public Health, Boston, MA 02115, USA; bcoull@hsph.harvard.edu

**Keywords:** mixtures, combined exposures, environment, statistics, methods, risk assessment, health impact, epidemiology, chemicals, chemical interactions, non-chemical stressors, exposomics

## Abstract

Humans are exposed to a diverse mixture of chemical and non-chemical exposures across their lifetimes. Well-designed epidemiology studies as well as sophisticated exposure science and related technologies enable the investigation of the health impacts of mixtures. While existing statistical methods can address the most basic questions related to the association between environmental mixtures and health endpoints, there were gaps in our ability to learn from mixtures data in several common epidemiologic scenarios, including high correlation among health and exposure measures in space and/or time, the presence of missing observations, the violation of important modeling assumptions, and the presence of computational challenges incurred by current implementations. To address these and other challenges, NIEHS initiated the Powering Research through Innovative methods for Mixtures in Epidemiology (PRIME) program, to support work on the development and expansion of statistical methods for mixtures. Six independent projects supported by PRIME have been highly productive but their methods have not yet been described collectively in a way that would inform application. We review 37 new methods from PRIME projects and summarize the work across previously published research questions, to inform methods selection and increase awareness of these new methods. We highlight important statistical advancements considering data science strategies, exposure-response estimation, timing of exposures, epidemiological methods, the incorporation of toxicity/chemical information, spatiotemporal data, risk assessment, and model performance, efficiency, and interpretation. Importantly, we link to software to encourage application and testing on other datasets. This review can enable more informed analyses of environmental mixtures. We stress training for early career scientists as well as innovation in statistical methodology as an ongoing need. Ultimately, we direct efforts to the common goal of reducing harmful exposures to improve public health.

## 1. Introduction

All humans are exposed to a diverse array of chemicals across their lifetimes. Cumulatively, and at a given point in time, a person’s individual exposures constitute a mixture of compounds. Depending on where a person lives and their daily activities, that person is likely exposed to varying levels of trace elements, volatile organic compounds, pesticides (organophosphates, carbamates, pyrethroids, organochlorines), perfluoroalkyl and polyfluoroalkyl substances, polycyclic aromatic compounds, organophosphate ester flame retardants and plasticizers, phthalates, fine particulate matter, nutrients, pharmaceuticals, as well as non-chemical exposures such as temperature, psychosocial stress, nutrition, and residential neighborhood, workplace, or school conditions. Determining how mixtures of exposures impact human health is a longstanding priority of the National Institute of Environmental Health Sciences (NIEHS) and the environmental health research community. NIEHS supported workshops in 2011 and 2015, which provided insights into the limitations and opportunities for mixtures research and brought to light statistical methods considerations for data analyses [1,2]. Mixtures data used for analyses includes biomarkers of chemical exposures measured in biological and environmental samples as well as questionnaire-based information, air pollution monitoring, and geospatial data. It can be challenging to accurately analyze this data because variables are often highly correlated, include many missing observations (due to limits of detection for common chemical assays) or violate important assumptions of common statistical modeling strategies. A collective message following these NIEHS workshops was the need for cross-disciplinary collaboration, informed model selection, ongoing novel statistical methods development, and resource sharing. 

To specifically address the analytical challenges of environmental mixtures research, NIEHS launched a funding initiative in 2017, Powering Research through Innovative methods for Mixtures in Epidemiology (PRIME) [3]. The purpose of PRIME was to support the development of innovative statistical, data science, or other quantitative approaches for studying the health effects of complex environmental mixtures in epidemiology. The PRIME program funded six research project grants beginning in 2018. These projects resulted in an explosion of new methods now available for consideration and application. 

In this review, we summarize 37 new statistical methods supported by the NIEHS PRIME Program, with the goal of enabling understanding and application in real-world epidemiological investigations. These methods have appeared in the published literature (or in a pre-print server such as *arXiv* followed by peer-reviewed journal publications) but have never been summarized as an entire body of work. We aim to highlight considerable advancements from PRIME teams and provide a starting point for the research community to navigate these methods in a way that informs application. Due to the breadth of methods covered, we have provided a high-level overview of each method, leaving the statistical theory and related details for the independent manuscripts cited in this paper. To enable and encourage a common ontology for the comparison of methods, we follow strategies presented in previous review papers that categorize methods by research question(s) as well as critical features such as model goals and assumptions, application, types of data for which a method is applicable, and strengths and weaknesses. We also link to software to encourage application and testing on other datasets. 

## 2. Powering Research through Innovative Methods for Mixtures in Epidemiology (PRIME) Initiative

NIEHS launched a funding initiative in 2017, Powering Research through Innovative methods for Mixtures in Epidemiology (PRIME) [3]. The purpose of PRIME was “to stimulate the development of innovative statistical, data science, or other quantitative approaches to studying the health effects of complex chemical mixtures in environmental epidemiology,” by supporting de novo approaches, applications of quantitative methods used in other fields (i.e., those outside of epidemiology), and extensions or improvements to existing methods used in mixtures analyses [3]. Specific goals of PRIME were to improve existing methods to understand the complex relationships between NIEHS-relevant exposures and health outcomes, stimulate innovative interdisciplinary methods for mixtures in epidemiology, compare existing and novel methods including strengths and weaknesses across methods in various exposure/disease contexts, and develop tools and related resources such as new software. PRIME projects were required to be led by a researcher with expertise in a field such as statistics, mathematics, engineering, or related computational fields and involve an interdisciplinary team. These approaches were expected to account for known data complexities as well as the underlying biology of exposures to guide model approaches. Real world and simulated datasets were required for use by each team to evaluate method performance. This included the comparison of methods across large and small sample sizes, a range of different exposures and exposure correlation structures, and consideration of binary and continuous exposure and outcome measures. PRIME was also designed to facilitate cutting-edge interdisciplinary science to advance research in environmental mixtures in ways most relevant to NIEHS. 

## 3. Results: Key Advancements Offered by New and Expanded Methods

The PRIME application submission and scientific peer review meeting took place in July 2017. Six grants were awarded in early 2018 and are summarized in Table 1. Each project addresses several statistical challenges inherent in analyzing mixtures data and brings unique datasets for testing and evaluation of these methods. Study populations span longitudinal birth cohorts as well as cohorts of adults, cross-sectional studies, aggregated geospatial data, and educational data available in the public domain. Exposures evaluated include air pollution, metals, pesticides, flame retardants, persistent organic pollutants, and other endocrine disrupting chemicals. Some researchers proposed to examine how non-chemical exposures, like stress and nutrition, may amplify or protect against the adverse health effects of a chemical mixture. Health outcomes in children and adults include neurological, reproductive, cardiovascular, metabolic endpoints, child development, and educational outcomes. One project expanded to develop new methods for evaluating SARS-CoV-2 transmission and COVID-19 hospitalization in the United States, with a particular focus on spatiotemporal variation in air pollution exposures and viral transmission. 

In this review, we describe 37 de novo or expanded statistical methods for mixtures, related to the NIEHS-funded PRIME projects. We refer to these as “PRIME methods”, however, each method should be credited specifically to the authors of the corresponding publications. Most of these methods have accompanying R packages (or functions within existing R packages) to enable application. Compared to existing approaches at the time the PRIME program began (referred to here as “pre-PRIME methods”), each method offers unique advancements and advantages, which are summarized in Table 2 and presented in greater detail in the Supplementary Material (Table S1). 

**Figure 1:**
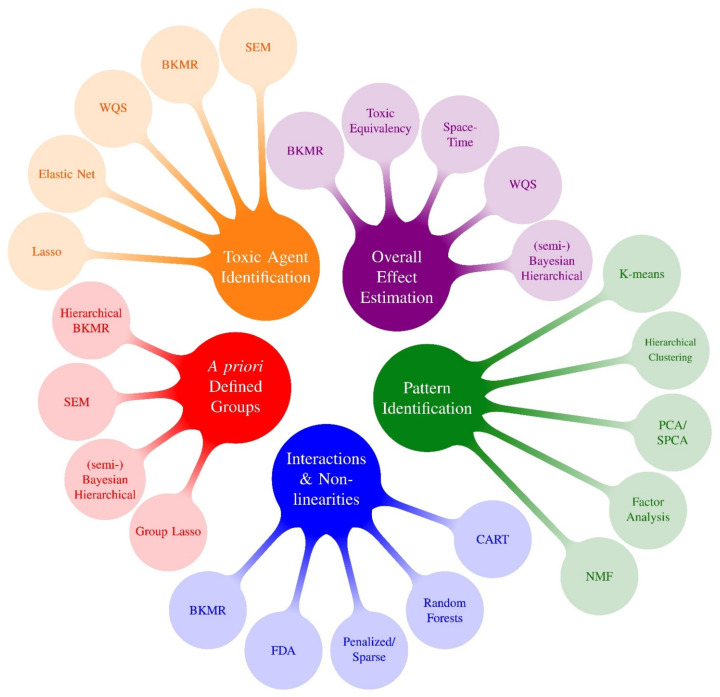
Mixtures Methods x Research Questions ^1^: Methods Preceding PRIME. ^1^ Research questions following Gibson et al., 2019 review: (1) Overall effect estimation: What is the overall effect of the mixture and what is the magnitude of association? (2) Toxic agent identification: Which congeners or chemicals are associated with the outcome? What congeners/chemicals are most important? (3) Pattern identification: Are there specific exposure patterns in the data? (4) A priori defined groups: What are the associations between an outcome and a priori defined groups of exposures? (5) Interactions and non-linearities: Are there interactions between exposures? Is the exposure-response surface non-linear? (6) Exposure-response relationship: What is the exposure-response relationship between each chemical and the outcome? Because almost all methods that investigate interactions also characterize potentially nonlinear exposure-response functions, we group questions #5 and #6 into a single bubble in this figure.

**Figure 2:**
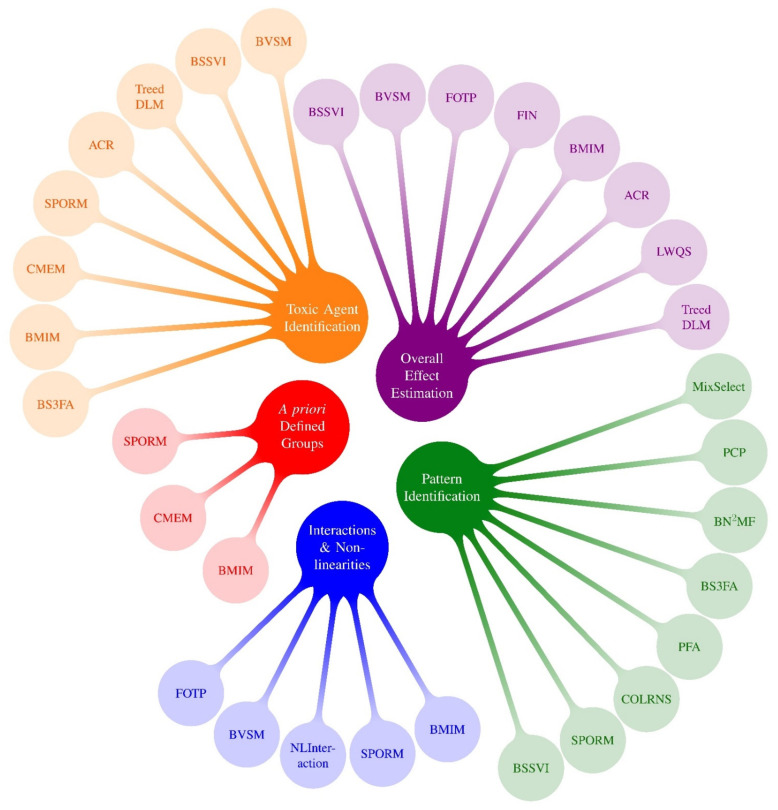
Mixtures Methods x Research Questions ^1^: Highlighted Methods from PRIME. ^1^ Research questions following Gibson et al., 2019 review: (1) Overall effect estimation: What is the overall effect of the mixture and what is the magnitude of association? (2) Toxic agent identification: Which congeners or chemicals are associated with the outcome? What congeners/chemicals are most important? (3) Pattern identification: Are there specific exposure patterns in the data? These can be managed with clustering and dimension reduction methods. (4) A priori defined groups: What are the associations between an outcome and a priori defined groups of exposures? (5) Interactions and non-linearities: Are there interactions between exposures? Is the exposure-response surface non-linear? (6) Exposure-response relationship: What is the exposure-response relationship between each chemical and the outcome? Because almost all methods that investigate interactions also characterize potentially nonlinear exposure-response functions, we group questions #5 and #6 into a single bubble in this figure.

Many of these pre-PRIME methods, as well as some recent methods developed in parallel to PRIME, have been described in recent reviews [42,43,44,45]. These reviews present useful information, including full descriptions of those approaches and logical examples of how one might go about selecting a method for a given problem. One meaningful way to digest and categorize methods is by specifying the primary research question(s) of interest. This can be helpful when determining which model(s) to use in an epidemiological application. The concept of organizing statistical methods by research questions was one of the take home messages from the 2015 NIEHS workshop [1] and was characterized in more detail in recent reviews [43,44]. With this strategy in mind, Figure 1 displays mixtures methods that existed prior to PRIME by research question (pre-PRIME methods), and Figure 2 presents an analogous categorization of a selection of new methods developed by PRIME (PRIME methods). Because methods can address more than one research question, Table 3 displays methods by research question as a matrix. In this section, we describe the selected PRIME methods that fall into the following five research question categories: (1) Overall effect estimation: What is the overall effect of the mixture and what is the magnitude of association? (2) Toxic agent identification: Which exposures are associated with the outcome? What exposures are most important? (3) Pattern identification: Are there specific exposure patterns in the data? (4) A priori defined groups: What are the associations between an outcome and a priori defined groups of exposures? (5) Interactions and non-linearities: Are there interactions among exposures, and if so, what patterns of effect modification are identified? Is the exposure-response surface non-linear? Since most methods address more than one research question, the categorization below should not be considered exclusive. 

### 3.1. Overall Effect Estimation 

A common goal of overall effect estimation is to quantify the total effect of a mixture without having to first estimate the effects of individual exposures. There are several pre-PRIME methods that can address overall effect estimation. These include, but are not limited to, index models, such as Weighted Quantile Sum (WQS) regression [46] and Quantile G-Computation (qGc) regression [47], and response surface methods such as Bayesian Kernel Machine Regression (BKMR) [48] and Generalized Additive Models (GAM) [49]. These methods have been described in detail and widely applied. Over half of the PRIME methods can be used for overall effect estimation (Table 3). These include (in alphabetical order by acronym) Acceptable Concentration Range model (ACR), Bayesian Regression Tree Pairs (Bayes Tree Pairs), Bayesian Data Synthesis (BDS), Bayesian Kernel Machine Regression-Causal Mediation Analysis (BKMR-CMA), Bayesian Kernel Machine Regression-Distributed Lag Model (BKMR-DLM), Bayesian Multiple Index Model (BMIM), Bayesian subset selection and variable importance for interpretable prediction and classification (BSSVI), Bayesian variable selection for understanding mixtures in environmental exposures (BVSM), Directed Acyclic Graphs (DAG) analysis, Bayesian Treed Distributed Lab Models (DLMtree), Factor analysis for interactions (FIN), Fast, optimal, and targeted predictions using parameterized decision analysis (FOTP), Graph Laplacian based Gaussian Process (GL-GPs), Computational improvements for Bayesian multivariate regression models based on latent meshed gaussian processes (GriPS), Lagged Weighted Quantile Sum regression (LWQS), Resolving rotational ambiguity in matrix sampling (MatchAlign), Multiple exposure distributed lag models with variable selection (Mult DLAG), Repeated holdout Weighted Quantile Sum regression (RH-WQS), Scalable Gaussian Process regression via Median Posterior Inference (SGP-MPI), and Total Explained Variation (TEV). Because of the large number of PRIME methods that can be used for overall effect estimation, we highlight only a few methods in this section and describe unique distinctions in other sections.

Traditional implementation of BKMR may be limited when considering causality, time-varying exposures, or computational efficiency in massive datasets. Several new PRIME methods expand on BKMR strategies to address these limitations: BKMR-CMA, BKMR-DLM, and BMIM. These continue to be useful for overall effect estimation of a mixture, but offer additional advantages, and are covered in more detail in other sections (see Section 4.2, Section 4.3 and Section 4.4). 

Weighted Quantile Sum (WQS) regression estimates the mixture effect (i.e., the joint action of the components) on a health outcome. It uses an empirically weighted index of chemicals, in quantiles, which is used as a parameter in a regression model. Two types of ensemble steps include bootstrap sampling of subjects and random subset selection of components, which is useful when the number of exposures exceeds the sample size. Expanding upon this approach, RH-WQS generalizes WQS regression to perform repeated holdout random data splits [30]. This method is most useful when interest focuses on quantifying uncertainty associated with estimates of the weights and association parameters or when the number of exposures is very large. 

A different approach to overall effect estimation draws from the genome-wide complex trait analysis (GCTA) method, common in genome-wide association studies to estimate heritability [50]. Chen et al. extended this method to the context of highly correlated mixtures of pollutants and non-normal data to develop the total explained variation (TEV) approach. TEV estimates the explained variation of an outcome by a set of mixture pollutants and can be applied to a large number of exposures when the effects of the exposures are weak, and the exposures are highly correlated [39,41]. This approach is similar to EigenPrism, which seeks to perform inference (i.e., construct confidence intervals) for (1) the error of a high-dimensional (*p* > n) regression estimator, (2) the linear regression noise level, and (3) the genetic signal-to-noise ratio of a continuous valued trait [51]. 

### 3.2. Toxic AGENT Identification (Variable Selection)

It is often of interest to identify those components of a mixture that are most toxic to human health and/or most predictive of the outcome of interest. To this end, appropriate methods need to be applied that can disentangle independent associations and characterize the exposure-response relationship between individual exposures in the mixture and the outcome. Approximately half of the methods addressing overall effect estimation can also be applied to identify specific toxic agents (Table 3), which is often referred to as “variable selection” in the statistical literature. We highlight a few PRIME methods in this section where variable selection is one of the primary goals. These include Bayesian variable selection for understanding mixtures in environmental exposures (BVSM) and Bayesian subset selection and variable importance for interpretable prediction and classification (BSSVI), which represent expansions of Bayesian-based variable selection strategies.

BVSM is a variable selection strategy using sparse summaries of a linear regression model. It is most useful when trying to select variables and provide uncertainty quantification for a linear model used to characterize the effects of exposure [34]. Kowal et al. used this Bayesian regression model to identify social and environmental covariates important for predicting educational outcomes. For BSSVI, Bayesian subset selection is used to collect and summarize all near-optimal subset models to provide a complete predictive picture. It is useful in the presence of correlated covariates, weak signals, and/or small sample sizes, where different subsets may be indistinguishable in their predictive accuracy [33]. Notably, both BVSM and BSSVI can also be used for overall effect estimation.

Methods highlighted in other categories that can also be used for toxic agent identification include ACR, Bayes Tree Pairs, BKMR-CMA, BKMR-DLM, Bayesian Matrix Completion for hypothesis testing (BMC), BMIM, Bayesian partially supervised sparse and smooth factor analysis (BS3FA), DAG analysis, DLMtree, FIN, GriPS, Heterogeneous distributed lag models (Het-DLM), Mult DLAG, Bayesian semiparametric regression with sparsity inducing priors (NLinteraction), SGP-MPI, State Informed Background Removal (SiBAR), and Semi-Parametric Odds Ratio Model (SPORM) (Table 3).

### 3.3. Pattern Identification

If the aim of the study is to inform the design and development of targeted interventions or policies/regulations, then first identifying common behaviors or sources that give rise to shared exposure profiles in the study population may be of interest. Because it is common for constituents of a mixture to be moderately to highly correlated, another important aspect of studying health effects of mixtures is learning low-dimensional structure in the data for interpretability and for improving statistical efficiency. For these purposes, exposure pattern identification methods can be used. This set of methods aims to identify common and consistent patterns in exposures that are shared across the population, which can be subsequently linked to adverse health outcomes. Pattern identification methods commonly incorporate some clustering or dimensionality reduction, as the identified patterns are usually fewer than the number of exposures in the mixture. A more flexible approach than assuming a simple group structure is to rely on principal components analysis (PCA), or the model-based alternative factor analysis (FA). FA supposes that there is a lower-dimensional set of independent factors underlying a moderate to high dimensional set of chemical exposures. By applying exploratory FA, one can learn the number of factors and how these factors relate to the measured exposures.

Exposure pattern identification methods can be unsupervised (i.e., the patterns are first identified independently of any health outcome and associations with different health outcomes are examined at a second stage) or supervised (i.e., patterns are identified specific to the outcome of interest). The latter can also provide information about specific biological pathways for the association of interest. Pattern identification methods that specifically consider exposures that vary over space and time are discussed in greater detail in Section 4.6.

Bayesian methods developed by investigators from the Duke University PRIME project represent the state of the art in exploratory FA and have generalized FA methods to account for information that is commonly available in epidemiology studies. The perturbed factor analysis (PFA) approach focuses on studying similarities and differences in exposure profiles across groups. For example, insights obtained from PFA can be used in studying environmental justice research applications. Roy et al. evaluated differences in exposure profiles across biological or social constructs of race/ethnicity [18]. Usual FA methods treat the different exposures as exchangeable a priori, while in the mixtures context there is typically information available on chemical class or related categories. Roy et al. included a comparison to Bayesian multi-study factor analysis [52], which can be implemented via the MSFA package [53]. The generalized infinite factor model (GIF-SIS) allows for the inclusion of such information in exploratory FA, while learning the number of factors and the loadings structure flexibly [13]. Schiavon et al. [13] compared performance with popular Bayesian FA methods based on the multiplicative gamma process [54], as implemented in the hmsc package [55]. 

One issue with interpreting results from exploratory FA is non-identifiability; for example, this leads to challenges in summarizing Bayesian posteriors. The resolving rotational ambiguity in matrix sampling (MatchAlign) method addresses the issue of rotational ambiguity in a wide class of Bayesian models that involve unidentifiable random matrices, such as the Gaussian factor model. In this setting, without identifiability constraints, reliable posterior summaries of model parameters cannot be obtained directly from the MCMC output. MatchAlign is also a computationally efficient post-processing algorithm that allows inference of non-identifiable parameters. The approach orthogonalizes the posterior samples using Varimax and then tackles label and sign switching with a greedy matching algorithm [19]. Poworoznek et al. [19] compare the proposed MatchAlign algorithm with methods in Papastamoulis and Ntzoufras [56]. 

An additional issue with usual exploratory FA models is the focus on learning linear lower-dimensional structure. To address this limitation, one can instead characterize the lower-dimensional structure as a smooth surface or “manifold”. The manifold reconstruction via Gaussian processes (MrGap) approach develops an approach for inferring such a manifold from noisy higher-dimensional data [17].

Principal Component Pursuit (PCP) is a pattern identification method used in computer vision applications. Gibson et al. adapted and extended PCP to the mixtures context [8]. PCP requires minimal assumptions, like Principal Components Analysis (PCA) [57] but is substantially different in several ways. First, it decomposes a matrix of exposures into a low-rank and a sparse matrix. The low-rank matrix contains information about commonly shared exposure events, i.e., consistent patterns across the study units of analysis (subjects, days, etc.). The sparse matrix contains unusual, unique, or extreme exposure events that cannot be explained by the consistent patterns in the low-rank matrix. Generally, extreme or outlying observations may be thrown out in statistical modeling strategies. PCP leverages these extreme points to add information to modeling in a way not previously done in environmental epidemiology. The method also offers flexibility to be applied to various settings. The investigators compared PCP to PCA in simulations. Through cross-validation, PCP identified the true number of patterns in all simulations, while PCA did so only in 32% of the simulations. In general, PCP outperformed PCA in most simulated scenarios [8]. 

Like PCP, Bayesian non-parametric non-negative matrix factorization (BN^2^MF) also aims to robustly identify exposure patterns. BN^2^MF also estimates the number of exposure patterns as one of the model parameters. Furthermore, BN^2^MF also provides confidence estimates around the estimated parameters, quantifying the model’s confidence in the estimation of these parameters [7].

Methods highlighted in other categories that can also be used for exposure pattern identification include BS3FA, BSSVI, FIN, FOTP, GIF-SIS, identifying main effects and interactions among exposures using Gaussian processes (MixSelect), SiBAR, and SPORM.

### 3.4. A Priori Defined Groups

In some research contexts, some information is already known about the mixture of interest. For example, it is possible that the exposures in the mixture can be organized in some hierarchy, e.g., similar chemical structure, and/or the researchers may a priori be interested in associations with pre-specified groupings of the mixture members, e.g., traffic-related air pollutants. In those cases, methods that can accommodate such a priori defined groupings are desirable. Defining groupings for this method requires a priori information on the included exposures. PRIME methods that can also use pre-specified structure among exposures include BKMR-CMA, BMIM, CVEK, GIF-SIS, and SPORM. Details of these methods are provided in other sections. 

### 3.5. Interactions and Non-Linearities 

Another major advancement from PRIME is refined methods for estimating and testing interactions within an exposure-response framework. Ferrari et al. developed Mixselect, which uses a Gaussian process to parameterize the multivariate exposure-response surface and partitions this surface into main effects and higher order interactions [16]. In closely related work, Antonelli et al. employed Bayesian sparsity priors with a semiparametric regression framework to produce variable importance scores for each exposure in the mixture as well as for each pairwise interaction (NLinteraction) [29]. Related work from Antonelli et al. used Bayesian variable selection to identify interactions among exposures experienced at the same or different points in time (i.e., different exposure windows) (MultDLAG) [22]. Liu et al. proposed a cross-validated ensemble of kernels (CVEK) that yields a formal hypothesis test of an interaction between two sets of exposures, such as two different mixtures representing nutrition and environment [24]. This work compared CVEK’s power to detect such interactions to that from several existing methods, such as interaction sequence kernel association test, a Gaussian kernel machine test, and a spline-based generalized additive model, via application to a common dataset and showed that CVEK can yield increased power to detect interactions relative to these existing methods.

Ferrari et al. also developed a latent factor joint model that incorporates shared factors in both the exposures and outcome/response components [12]. They applied quadratic regression in the latent variables of the response to induce flexible dimension reduction. The approach is referred to as Factor analysis for Interactions (FIN) and was applied to National Health and Nutrition Examination Survey (NHANES) data [12]. The FIN method is particularly useful when interested in interactions in high-dimensional settings. FIN can also be used to address overall effect estimation and toxic agent identification/variable selection. Ferrari et al. compared FIN with four competitors for high-dimensional interaction selection including penalized interaction estimation (PIE) [58], regularization algorithm under marginality principle (RAMP) [59], a framework for modeling interactions with a convex penalty (Family) [60], and a lasso for hierarchical interactions (HierNet) [61].

Methods highlighted in other categories that can also be used to address interactions or non-linearities include TEV, BSSVI, BVSM, MatchAlign, BDS, BKMR-CMA, GriPS, SGP-MPI, BMIM, GL-GPs, Bayes Tree Pairs, BKMR-DLM, DLMtree, SPORM, and FOTP.

## 4. Other Statistical Advancements for Mixtures

The diverse array of PRIME methods presented in this paper touch on many other aspects of statistical methodology or challenges of environmental mixtures data. In this section, we describe the alignment of methods to data science strategies (e.g., data preparation and data architecture), exposure-response estimation, considerations of exposure timing (critical windows of susceptibility), inclusion of toxicity or related chemical information, and spatiotemporal variation in exposure. Finally, we note methods that are particularly useful for high dimensional and noisy data, risk assessment, and improvements in model performance, efficiency, and interpretation. As in the previous section, the methods discussed are not comprehensive, and many methods are relevant to more than one category or section. This information may also be useful to aid the selection of method(s) to apply to epidemiological datasets.

### 4.1. Data Science and Data Preparation Strategies

New exposure science and related technologies enable the generation of terabytes of data, available to researchers for analysis. However, these datasets are often generated by unlinked parallel streams in varying data formats. An example approach addressing these challenges was developed by the Notre Dame/Rice project, in which population-level datasets were linked at the individual level using iterative matching techniques. A specific method described by Feldman et al. is Bayesian Data Synthesis (BDS) [32]. This method provides a framework to simulate fully synthetic datasets of mixed data types. This method is useful when a dataset cannot be shared publicly, but analyses on it are published. The dataset may be comprised of mixed categorical, binary, count, and continuous datatypes. It can handle missing data and provides customized metrics for attribute risk disclosure and other privacy concerns. The longitudinal, linked, and spatial data have allowed for the detection of subtle outcome disparities, improved causal inference, and identified key drivers of disparities [62]. 

Not to be forgotten are the important considerations of data management, imputation of missing data, and related data preparation prior to analyses. The imputation of multivariate data by normal model (MVNimpute) method addresses the common issue of missing and censored data with a new imputation approach that can simultaneously impute data that are missing or censored by limits of detection [38].

### 4.2. Estimation of the Exposure-Response Surface

Response surface methodology seeks to estimate the multivariate exposure-response surface that describes the relationship between an outcome and a matrix of exposures. This includes flexible estimation of the form of the association (linear, quadratic, etc.) between each exposure and the outcome, as well as any interactions between exposures in their effects on the outcome. These types of methods are popular in mixtures research and many are within the broad domain of nonparametric or semiparametric regression models. Prior to the PRIME program, prominent examples were multivariate generalized additive models (GAMs) and Bayesian kernel machine regression (BKMR). 

The PRIME program has developed novel new methods that flexibly estimate a multivariate exposure-response relationship using latent variables as well and kernel machine-based methods. Ferrari et al. developed Bayesian factor analysis models (FIN) that do well modeling pairwise and higher-order interactions among many variables (see Section 3.5), and because it parameterizes the full exposure-response relationship to be one implied by the association between the outcome and a smaller number of factors, a full exposure-response surface can be estimated by the method [12]. Ferrari et al. followed this up with MixSelect, which decomposes a Gaussian process regression, a form of kernel regression with a Gaussian kernel, into main effects and interaction components [16]. These estimates could also be used to obtain an overall exposure response relationship. Ferrai et al. compared MixSelect with BKMR, Family, HierNet, PIE and RAMP methods.

One disadvantage of existing approaches is the difficulty in visualizing and ultimately interpreting the multivariate surface when the number of exposures is high. The factor analysis work of Ferrari et al. noted above addresses this issue by using factor analysis to reduce the dimension of the exposure space [12]. McGee et al. proposed another approach, BMIM, to address this weakness [5]. BMIM combines the strengths of existing exposure-index methods, such as weighted quantile sum (WQS) regression and qGc, by reducing the dimensionality of the exposure vector and estimating index weights with variable selection and treating these indices as inputs into the kernel regression framework. Because the number of indices is typically much smaller than the number of exposures, interpretation is simpler. BMIM was compared to two existing methods, BKMR (a special case) and qGc (an index model), using simulated data as well as application to NHANES data on associations between persistent organic chemicals and leukocyte telomere length, a dataset previously used for comparing mixtures methods [44]. BMIM also has the advantage that it allows for the incorporation of auxiliary toxicological information such as toxic equivalency factors into an analysis through the use of informative priors for the index weights [63].

Other methods that address exposure-response estimation include NLinteraction, BKMR-CMA, MrGap, SGP-MPI, ACR, GL-GP, BKMR-DLM, DLMtree, and SPORM.

### 4.3. Timing of Exposures and Periods of Susceptibility

In addition to the analysis of environmental mixtures, another critical issue in environmental health is identifying windows of susceptibility. For example, it has been well-documented that gestation can be a critical window of susceptibility for the developing fetus, and this level of susceptibility can differ from that during childhood and adolescence. Accordingly, longitudinal epidemiological cohorts such as birth cohort studies typically measure exposures of interest across multiple developmental windows [64]. There now exists a suite of methods to estimate critical windows of susceptibility from such data for single chemicals. These include distributed lag models (DLM) [65], multiple informant models, and clustering analysis of exposure trajectories [66]. 

Because individuals are rarely exposed to single chemicals in isolation, the issue of critical windows of susceptibility extends to mixture epidemiology. Prior to the initiation of the PRIME program, most methods for evaluating the association between mixtures and an outcome were designed for studies with exposures measured at a single or discrete time points [64]. Notable exceptions were lagged weighted quantile sum (LWQS) regression [67], which uses a weighted quantile sum (WQS) regression within a DLM framework when a mixture is measured with high temporal resolution over time, and lagged kernel machine regression (LKMR) [68], which extends kernel machine regression to the setting in which a mixture is measured at a small number of discrete timepoints (e.g., trimesters during pregnancy). 

The PRIME projects have expanded the statistical toolbox for estimating critical windows of susceptibility for a mixture in several ways. Gennings et al. extended lagged WQS regression to accommodate a large number of exposures (LWQS using the random subset selection ensemble step), which often occurs in exposomic research and where the exposure timing may vary across subjects [28]. Wilson et al. embedded a distributed lag structure within a kernel machine regression (BKMR-DLM) to analyze data on multiple exposures measured repeatedly over time (e.g., weeks during pregnancy) [23]. This work compared the performance of BKMR-DLM to additive distributed lag and distributed lag nonlinear models via simulation and showed that BKMR-DLM can improve upon existing methods in estimating the exposure-response function when this function is non-additive. Antonelli et al. introduced multiple exposure distributed lag functions (MultDLAG), a method to identify time-dependent interactions among distributed lags for multiple exposures [22]. This approach allows investigators to assess whether exposure in one developmental window can either increase or decrease susceptibility to exposures experienced in subsequent windows. 

Mork et al. presented two relevant strategies to address exposure timing. A regression tree-based model (Bayes Tree Pairs) can be used to represent distributed lag functions for mixtures of exposures observed at high temporal resolution (e.g., weeks during pregnancy) [25]. The approach uses an additive ensemble of tree pairs that defines structured main effects and interactions between time-resolved predictors and performs variable selection to select out of the model predictors not correlated with the outcome. This approach is computationally efficient, which is an advantage over some of the other flexible distributed lag models for mixtures for large datasets. Mork et al. extended this approach to allow for nonlinear distributed lag models (DLMtree), which does not restrict the association between the outcome and an exposure experienced at any given time to be linear [26]. Both papers compared via simulation these tree-based methods to several established spline-based distributed lag models, and the first paper also included a recently developed critical window identification method to the comparisons. Results suggested that the tree-based methods were competitive to, or improved upon, existing methods in terms of estimation of the distributed lag functions in several scenarios and yielded low false positive rates for critical window selection. An additional extension addresses the heterogeneity of the associations between critical windows of exposures and outcomes (e.g., across different levels of individual, family, or neighborhood characteristics) in high dimensional datasets (HetDLM) [27]. HetDLM can be particularly useful for identifying susceptible subgroups of populations. Although this initial example of HetDLM is applied to single exposure data only, current work by PRIME investigators focuses on extending this method to mixture scenarios. 

Kowal et al. developed a fast, optimal, and targeted predictions (FOTP) algorithm based on a functional Bayesian approach to address the variable impact of maternal air pollution exposure during pregnancy on educational outcomes of children. In general, given a target (or functional) of interest and a Bayesian model, it addresses how one can construct accurate, simple, and efficient predictions. This method is most useful when the measured outcome can be represented as a functionality of the exposure variables and the goal is either to predict or interpret the multiple exposures on outcomes or functionalities of the outcomes [35]. 

### 4.4. Epidemiological Methods and Causal Models

Epidemiologic studies of a mixture need to distinguish predictive models and causal models. The latter is usually the goal of environmental epidemiologists. While predictive models can rely on purely statistical criteria for selection of variables for inclusion in a model, causal models must carefully consider which variables to include as exposures, confounders, effect measure modifiers, or mediators. The choice relies on subject matter knowledge, often embedded in directed acyclic graphs (DAGs) for distinguishing between confounders, mediators, and colliders. As a simple example, suppose there are two exposures of interest. In some situations, including both in a regression model (the underlying basis for most mixtures methods) could increase bias of individual components compared with examining one variable at a time [6]. On the other hand, it may decrease bias of overall measures of effect [69]. Careful consideration of DAG construction for the context of mixtures models is an important step to consider prior to statistical model selection.

DAGs are also essential for the implementation of a causal mediation analysis, in which interest focuses on whether a part or whole of an exposure effect is mediated through a hypothesized pathway represented by a mediating variable. Such knowledge is important for the design of effective interventions to prevent adverse effects of exposure. Devick et al. [4] proposed methodology to estimate the natural direct effect (NDE), natural indirect effect (NIE), and controlled direct effects (CDEs) of a complex mixture exposure on an outcome through a mediator variable (BKMR-CMA). This approach applied BKMR to allow for all possible interactions and nonlinear effects of (1) the co-exposures on the mediator, (2) the co-exposures and mediator on the outcome, and (3) selected covariates on the mediator and/or outcome. 

The University of Illinois Chicago project developed a network modeling approach (SPORM) to investigate the complex association among outcomes, intermediate biological markers, and mixtures of pollutants applicable to mixed discrete and continuous data types. It is most useful when the relationship among different types of variables is of major interest [14,39]. SPORM models association by odds ratio functions and can be applied to answer four of the five research questions discussed in Section 3 (Table 3). SPORM can be used to model the relationships between groups of variables without modeling the relationship within each group, offering more flexibility compared to generalized linear models. 

### 4.5. Toxicity and Related Chemical Information 

Toxicology and pharmacology have a long history of interest in “additivity” and interactions between chemicals with methods falling into two general classes: methods for studying whole, well-defined mixtures and component-based methods that predict an overall effect based on the individual dose-response curves of the mixture components plus models of non-interaction (note that “interaction” and “additivity” can have different meanings in toxicology, epidemiology, and statistics). Such predictions can be represented as response surfaces, representing a connection to response-surface methods in epidemiology that try to estimate the response surface from data points (see Section 4.2). Relative potency factors—e.g., the Toxic Equivalency Factors (TEFs) of dioxin-like compounds—are one of the more familiar models of non-interaction/“additivity” in toxicology. One promising line of research connecting mixtures toxicology and epidemiology is the use of animal or in vitro data as priors for epidemiological studies using Bayesian methods such as BMIM [5].

Another method incorporating chemical information into statistical models is Bayesian Matrix Completion for hypothesis testing (BMC) [10]. BMC applies Bayesian inference about chemical activity on mean and variance dose-response measurements, while accounting for sparsity of data. The method is best used when there are large amounts of missingness in mixtures data, the user wishes to predict the activity of a chemical pair, the dose-response shapes are non-linear, and/or heteroscedastic errors are evident. This approach has been demonstrated to predict toxicity and health endpoints in the ToxCast/Tox21 data but has not yet been applied in epidemiological datasets.

Moran et al. developed Bayesian partially supervised sparse and smooth factor analysis (BS3FA) [11], which applies Bayesian inference to link chemical molecular structure to dose response. By fitting the model to ToxCast/Tox21 data, a distance between chemicals can be learned, which is targeted to a particular toxicity endpoint. One can predict dose response with uncertainty quantification for any chemical-assay pair (*i*,*j*) by borrowing information across (a) chemicals having a similar molecular structure to chemical *i* and/or (b) dose response data for chemical *i* for assays $j^{\prime }\neq j$. Chemical activity profiles across assays can even be predicted for chemicals lacking any in vivo or in vitro testing data. This provides invaluable prior information for use in epidemiological analyses of mixtures, as many/most mixture constituents to which humans are exposed have little or no direct in vivo or in vitro testing data available. 

### 4.6. Spatiotemporal Methods

Leveraging the unique data architecture noted in Section 4.1, several new methods address the spatiotemporal variability in distributions of exposure. The State Informed Background Removal (SiBAR) approach quantifies ‘background’ versus ‘source-influenced’ contributions to air pollutant time series. It applies a hidden Markov model to determine what background levels of pollutants can be measured across a geographic area (e.g., heavy to moderately polluted urban area). It is most useful when interested in apportioning pollutants to local sources [37]. A separate method, the spatiotemporal case-crossover (SCC) provides a strategy for the case-crossover study design in a spatial-temporal setting. It incorporates a temporal case-crossover and a geometrically aware spatial random effect based on the extended Hausdorff distance. This method is most useful when information is available for irregular spatial regions (e.g., census blocks) over time and when the constant exposure assumption is not reasonable [36]. Although this initial example of SCC is applied to ozone data only, the method can be used more broadly in a variety of mixtures data types.

Increasingly massive spatiotemporal datasets are collected on exposures, as well as on environmental covariates that relate to exposures and/or health outcomes. In air pollution monitoring, many people in the public have installed PurpleAir monitors at their homes; these “crowd-sourced” data can be used to augment better-calibrated EPA monitors that have much lower spatial coverage. It is of critical interest to fit accurate models of the level of exposure to different pollutants at each spatial location at different times while realistically characterizing uncertainty in such predictions; such data can be coupled with health outcome data in relating air pollution to health outcomes, including complex infectious disease outcomes—e.g., related to COVID-19. The Duke project has been developing frameworks for flexible and computationally scalable spatiotemporal modeling, designed to capture the complexities in both air pollution and health outcome data. Flexible spatiotemporal models typically rely on variations of Gaussian processes (GP); such models face computational bottlenecks as data size increases. Spatial Multivariate Trees (SPAMTREE) address the challenge of modeling huge multivariate data, while accommodating multiresolution sensor data [20]. A related approach focused on improving computation for Bayesian multivariate regression with spatial random effects is GriPS [15]. For aquatic pollutants, exposure data are constrained to fall in a restrictive domain, corresponding to the locations of rivers, lakes, or under-ground water bodies. Usual GP models do not accommodate such a restrictive domain, and face inaccuracies in prediction, particularly when data are sparse. Graph Laplacian-based GPs (GL-GPs) solve this problem, learning a covariance function that respects the restrictive domain of the data [14]. In related work, Jin et al. developed a Bag of DAGs (BAG) approach for scalable modeling of non-stationary spatiotemporal process, with a particular motivation to modeling of air pollution data that may have directional dependence due to prevailing winds [9].

### 4.7. Risk Assessment and Regulatory Relevance

An important challenge of mixtures research is direct translation to regulatory decision making. To address this, Gennings et al. recently described a new class of models that include the regulatory concept of acceptable concentration range (ACR) [21]. ACR is a new class of nonlinear statistical models for human data that incorporate and evaluate regulatory guideline values into analyses of the health effects of exposure to chemical mixtures. The ACR model allows for human data to suggest points of departure (PODs) for comparison to in vivo estimates from single chemicals. The method can be used to estimate PODs from human data which may suggest data-driven uncertainty factors (i.e., so-called mixture assessment factors (MAFs)) in risk assessments of single chemicals. This model also relates to considerations around the exposure-response function applied to outcome-chemical concentrations (see Section 4.2). 

### 4.8. Model Performance, Efficiency, and Interpretation

Some existing statistical methods for analyzing the health effects of environmental mixtures were motivated by data collected in moderately sized toxicology studies or epidemiologic cohort studies. Accordingly, the fitting algorithms do not necessarily scale to big data settings, such as those that are commonly encountered when interest focuses on analyzing electronic health records or other administrative databases. For instance, standard kernel regression methods, such as Gaussian process regression and BKMR [48,70] involve calculations on matrices of size n × n, where n is the number of observations in the data. These calculations get prohibitively computationally expensive for large samples. Several projects in the PRIME program have focused on computational strategies for scaling up statistical methods for environmental mixtures to big data settings, thereby broadening the applicability of these methods. For instance, Peruzzi et al. have developed meshed Gaussian process models for scalable inference [71]. This approach is applicable to Gaussian kernel methods similar to those employed by BKMR as well as to spatiotemporal problems described in Section 4.6. Sonabend et al. described a split-and-conquer strategy that applies BKMR to sub-samples of the full population and combines the results from these smaller analyses in such a way that it obtains inferences comparable to those one would obtain if it were possible to analyze the data all at once [31].

## 5. Software

Essential for application, PRIME methods are available in open-source software, primarily R packages or functions within existing R packages. A few methods were developed in SAS and can be implemented with shared SAS code. Many methods can be retrieved from the NIEHS PRIME GitHub (https://github.com/niehs-prime/, accessed on 21 December 2022) or individual GitHub sites. Links to all available open-source code are provided in the Supplementary Material (Table S1). 

## 6. Discussion

Over the last decade, the growth of complex, correlated, and diverse environmental data has increased the demand for robust and versatile statistical methods to determine the association between mixtures and health outcomes. The NIEHS PRIME program investigators and colleagues have contributed a remarkable collection of methods to address this demand. In this paper, we discuss 37 new methods from PRIME, and include links to the available software and documentation to enable broader application. Additional methods are forthcoming, from PRIME projects as well as independent research efforts for mixtures, exposomics, and broader data science domains. With new methods developed, testing and evaluation through real-world applications are important next steps.

Application can only be possible with an equipped work force. To leverage the resources provided by PRIME, students and new investigators in epidemiology and environmental health need proficiency in R programming, statistical methods, epidemiology, and toxicology. Training within each of these fields is already dense and typically tailored to sub-specialty areas. Independent workshop training such as the Columbia University Mixtures Workshop [72] and pre-meeting events for annual research conferences, can complement standard curriculum offered by institutions to fill in gaps of understanding in statistical methods for mixtures. With this approach, expansion to current curriculum offerings could consider some new PRIME methods presented here. 

This diverse collection of methods considers five research questions to guide model selection. However, organizing the methods by research questions (Figure 1 and Figure 2, Table 3) is not the only approach to enable digestion of Table 2 and direction of model selection. For example, Taylor et al. classified methods according to their function, e.g., variable selection, classification and prediction, etc. Although selecting methods according to research question best guides method selection based on research needs, other factors should also be considered, such as the amount of data (number of observations), dimension of the data (number of outcomes and/or predictor variables), structure of the data (e.g., spatial correlations or repeated measures), study design, and others [1]. For optimal method selection, given the research question, all these factors should be considered. However, when users are guided by research questions, they have a handful of methods to consider for the purposes of their study and the specific details of methods can be more easily compared. 

The PRIME program has been incredibly productive, as seen in Table 2 and the descriptions of the developed methods therein. However, there is more to be done. These methods are extremely valuable, but only some of them apply to the many different types of outcomes such as counts or time to event (survival) endpoints. The PRIME program has brought a causal lens to environmental mixtures epidemiology, and continued work in this area would help ensure existing and new causal methods are widely applicable in mixtures research. As new technologies continue to generate biomedical data, on both exposure and health, at unprecedented dimension and volume, more needs to be done to develop reproducible and efficient methods for data fusion and integration of high-dimensional data on both exposure and health. The burgeoning area of Precision Environmental Health may also be appropriate to consider for application and expansion of these methods, where detailed individual-level exposure and covariate information can be used to guide health decision making and prevention strategies. The large body of work generated by this PRIME program promises to serve as an excellent foundation for future work on these and other critical problems in environmental health and epidemiology. 

As noted in Gibson et al., some (e.g., BKMR, WQS, qGc), but not all, pre-PRIME methods were specifically developed with environmental mixtures data in mind [44]. The PRIME methods described here offer a unique advantage to previous methods in that they were developed within the mixtures context. However, approaches and resources presented here are not exclusive to environmental health. Rather, PRIME methods can also be considered in other research fields in which interest focuses on the individual and joint impacts of many risk factors for an outcome (e.g., ‘omics, health policy, health disparities research, among others).

A broader goal of this work is to provide the best statistical tools for accurate research translation to public health decision making. National human biomonitoring data provides evidence of the wide range of exposures across chemical classes, locations, and across the lifespan. Of particular concern are populations with a disproportionately higher level of exposure and comorbidities that may act jointly or even synergistically to affect health. PRIME methods include those focused on exposure timing in prenatal/postnatal periods and the impact on health and development. Methods that incorporate geospatial information have also been developed or enhanced to identify communities at a higher risk of specific environmental exposures. Some of the PRIME methods address a research question around the overall effect estimation of the mixture, which often includes an estimation of the mixture effect as a measure of the joint action of the chemicals which, for example, may be acting along an adverse outcome pathway (AOP). Recent regulatory focus is on grouping chemicals, perhaps based on evidence of a common AOP, with potential regulatory decisions based on the group. Many of the PRIME methods can be readily extended to cases where the number of components exceeds the sample size (i.e., *p* > N). This is particularly important for data from untargeted assays of exposure which may provide evidence of emerging chemicals of concern, not just those “under the lamp post.” Regulatory decisions may include banning adverse chemicals in total or provide guidelines for exposure concentration ranges. PRIME methods may be useful for analysis of human exposure data to inform analytics for regulatory decision making by complementing standard risk assessment strategies and thereby improve public health recommendations.

## 7. Conclusions

We summarize a diverse collection of statistical methods for mixtures now available for broader application. This work compliments earlier reviews and provides a useful starting point for researchers considering analytical strategies for complex datasets. The short-term goal of this review paper is to enable broader dissemination and education on the available methods and encourage application in environmental epidemiology. Longer term, there is opportunity to apply methods to other scientific research communities, including but not limited to, data science, exposomics, and other ‘omics domains. These methods may also be applicable to the burgeoning efforts in Precision Environmental Health, expected to encompass massive amounts of exposure and covariate data across individuals at different time points. Training and early career development opportunities in mixtures remain essential. Ongoing “outside-the-box” thinking with regards to methods and interdisciplinary collaborations is critical, pursuing the common goal to reduce harmful exposures and improve public health.

## Figures and Tables

**Table 1:** Summary of PRIME Projects.

Project (Institutions(s)) ^1^	Summary	Exposures ^2^	Study Populations ^3^
Development and testing of response surface methods for investigating the epidemiology of exposure to mixtures (BU/Harvard)	Combines aspects of response surface modeling with index methods into the Bayesian Multiple Index Method (BMIM) and incorporates toxicological information. Special cases are a single index model and a full response surface of all exposures as in BKMR.	Dioxin-like compounds, PCBs, phthalates, parabens, bisphenols triclosan, UV filters, BFRs, PBDEs	RCC, EARTH
Principal Component Pursuit to assess exposure to environmental mixtures in epidemiologic studies (Columbia)	Adapts the method Principal Component Pursuit (PCP), used in computer vision applications, to the epidemiologic setting of mixtures of environmental pollutants.	PCBs, metals, air pollution	CHDS, CCCEH, SHS, SPARCS
Structured nonparametric methods for mixtures of exposures (Duke)	Incorporates chemical structure data and mechanistic constraints into nonparametric Bayesian regression methods to improve stability, performance, and interpretation in estimating dose response. Supplemental funding develops Bayesian modeling frameworks for including exposures in epidemiological models of infectious disease spread, as well as flexible spatiotemporal modeling with applications to study exposure effects on COVID-19 hospitalizations.	Phenols, OPs, perchlorate, PFCs, phthalates, BFRs, PAHs, pyrethroids, air pollutants	MSSM, NHANES, CHAMACOS, CLEAR, CDC COVID Data Tracker, NYTimes COVID Data, State Population by Characteristics
Methods for data integration and risk assessment for environmental mixtures (MSSM/Harvard)	Integrates temporally resolved exposure into models, evaluates how early (“priming” or “protective”) exposures can impact susceptibility to later exposures, and estimates regulatory guideline values for mixtures.	Tooth metal biomarkers; EDCs, dietary data	Colorado birth data; SELMA
Bringing Modern Data Science Tools to Bear on Environmental Mixtures (Notre Dame/Rice)	Develops data architecture to capture complex spatial location data for families, environmental exposures, and social stressors that vary over time. Leverages modern data science by applying rapidly evolving techniques for architecting data combined with hierarchical Bayesian models with variable selection, spatial models, and machine learning algorithms to large-scale environmental mixture and social exposure datasets of direct importance to child outcomes.	Air pollution, lead, social stressors	Aggregate North Carolina birth records, blood lead surveillance data, and educational system data to social and environmental exposures
Innovative Methodologic Advances for Mixtures Research in Epidemiology (UI Chicago)	Adapts genomics approaches to evaluate the total main effects and interactions of chemical exposures. Applies novel multivariate models for analyzing the complex relationship between health outcomes, biological intermediates, and environmental pollutants.	POPs, PCBs, OCPs, BFRs, PFCs, dioxins, heavy metals	NHANES, GLFCS, HCHS/SOL

^1^ Listed in alphabetical order, by institution. Project details available at NIH RePORTER: https://reporter.nih.gov/, accessed on 21 December 2021. Institutions: Columbia University Mailman School of Public Health, University of Illinois Chicago, Icahn School of Medicine at Mount Sinai, Harvard T.H. Chan School of Public Health, University of Notre Dame, Rice University, Boston University School of Public Health, Duke University. ^2^ BFRs: Brominated Flame Retardants; EDCs: Endocrine Disrupting Chemicals, OCPs: Organochlorine Pesticides; OPs: Organophosphorus Pesticides; PAHs: Polycyclic Aromatic Hydrocarbons; PBDEs: Polybrominated Diphenyl Ethers; PCBs: Polychlorinated Biphenyls; PFCs: Perfluorinated Chemicals; POPs: Persistent Organic Pollutants; UV: Ultraviolet. ^3^ CCCEH: Columbia Center for Children’s Environmental Health; CDC COVID Data Tracker: https://covid.cdc.gov/covid-data-tracker/#variant-proportions and https://data.cdc.gov/Vaccinations/COVID-19-Vaccinations-in-the-United-States-Jurisdi/unsk-b7fc, accessed on 21 December 2021; CHAMACOS: Center for the Health Assessment of Mothers and Children of Salinas; CHDS: Child Health and Development Studies; CLEAR: Climate Change, Environmental Contaminants and Reproductive Health; EARTH: Environment And Reproductive Health cohort; GLFCS: Great Lakes Fish Consumption Study; HCHS-SOL: Hispanic Community Health Study/Study of Latinos; MSSM: Mount Sinai Children’s Environmental Health Study; NHANES: National Health and Nutrition Examination Survey; NYTimes COVID Data: https://github.com/nytimes/covid-19-data, accessed on 21 December, 2021 RCC: Russian Children’s Cohort; SELMA: Swedish Environmental Longitudinal Mother and child, Asthma and allergy study; SHS: Strong Heart Study; SPARCS: NY Statewide Planning and Research Cooperative System; State Population by Characteristics: published by the U.S. Census Bureau breaks down 2019 U.S. state populations by Age. From Single Year of Age and Sex Population Estimates: 1 April 2010 to 1 July 2019—CIVILIAN (SC-EST2019-AGESEX-CIV) https://www.census.gov/data/tables/time-series/demo/popest/2010s-state-detail.html, accessed on 21 December 2021, WAS: Wisconsin Angler Study.

**Table 2:** PRIME Methods and Software.

Project ^1^	Method Acronym	Method Title	Summary	**Reference**
BU/ Harvard	BKMR-CMA	Bayesian Kernel Machine Regression-Causal Mediation Analysis	Performs a causal mediation analysis when exposure within the mediation framework is a mixture. Estimates a multivariate exposure response surface in a model for the mediator given exposure, and another for the outcome given the mediator and the outcome, both using BKMR.	[4]
BU/ Harvard	BMIM	Bayesian Multiple Index Model	Unifies exposure index models with the response surface method BKMR, allowing a spectrum of intermediate models of multiple indices. Models non-linear, non-additive relationships between indices and an outcome. Special cases are a single exposure index and a response surface of all exposures.	[5]
BU/ Harvard	DAG analysis	Use of causal methods for determining which exposures to include in a model	Applies directed acyclic graphs (DAGs) to determine inclusion of exposure variables. In some circumstances, including an exposure variable can increase bias. Determines causal relationships between exposures (or groups of exposures) and a health outcome.	[6]
Columbia	BN2MF	Bayesian Non-parametric non-negative Matrix Factorization	Matrix factorization that provides non-negative (and more interpretable) solutions for factors and loadings and uncertainty estimates for the estimated parameters. Used for exposure pattern identification, similar to PCP.	[7]
Columbia	PCP	Principal Component Pursuit	Unsupervised robust exposure pattern identification. Decomposes exposure matrix into a low-rank matrix (consistent patterns) and a sparse matrix (unique exposure events). Robust exposure pattern identification.	[8]
Duke	BAG	Bag of DAGs	A computationally efficient method to construct a class of non-stationary spatiotemporal processes in point-referenced geostatistical models. Accounts for uncertainty in directions of association over space and time by considering a mixture of direct acyclic graphs (DAGs)	[9]
Duke	BMC	Bayesian Matrix Completion for hypothesis testing	Bayesian inference about chemical activity on mean and variance of dose-response measurements accounting for sparsity of data. Used to characterize chemical activity and its uncertainty.	[10]
Duke	BS3FA	Bayesian partially supervised sparse and smooth factor analysis	Bayesian inference on how chemical structure relates to variation in dose-response measurements. Addresses how to jointly model structural variability in molecular features of a chemical and its dose-response profile.	[11]
Duke	FIN	Factor analysis for interactions	Bayesian factor analysis for inference on interactions. Estimates interactions between highly correlated chemical exposures and effect on health outcomes.	[12]
Duke	GIF-SIS	Generalized infinite factor model	Shrinkage prior to the loadings matrix of infinite factor models that incorporate meta covariates to inform the sparsity structure and has desirable shrinkage properties. Addresses how to incorporate a priori known structure among variables when fitting a member of the broad class of factorization models.	[13]
Duke	GL-GPs	Graph Laplacian based Gaussian Process	Gaussian process model with a covariance function that respects the geometry of highly restricted or nonlinear domains. Develops a covariance function for nonparametric regression that respects the intrinsic geometry of the domain without sacrificing computational tractability.	[14]
Duke	GriPS	Computational improvements for Bayesian multivariate regression models based on latent meshed gaussian processes	Computational improvements for Bayesian multivariate regression models based on latent Meshed Gaussian Processes. Addresses how to efficiently solve the big-n problem for GPs when the number of outcomes is large.	[15]
Duke	MixSelect	Identifying main effects and interactions among exposures using Gaussian processes	Identifies main effects and interactions among exposures using Gaussian processes. Addresses how to model potentially non-linear effects and high-order interactions of chemical exposures on health outcomes.	[16]
Duke	MrGap	Manifold Reconstruction via Gaussian Process	Local covariance Gaussian process model for estimating a manifold in high dimensional space from noisy data. Conducts inference on a low-dimensional, nonlinear manifold in high dimensional space when data are subject to measurement error.	[17]
Duke	PFA	Perturbed factor analysis	Factor analysis that captures common structure among groups of related observations. Distinguishes shared and group-specific covariance structure and expresses shared structure via a set of shared factors.	[18]
Duke	MatchAlign	Resolving rotational ambiguity in matrix sampling	Efficiently resolving rotational ambiguity in Bayesian matrix sampling with matching. Does inference on unidentifiable random matrices.	[19]
Duke	SPAMTREE	Spatial Multivariate Trees	Bayesian multivariate regression methods for big data using sparse treed Gaussian processes. Jointly models several imbalanced variables flexibly and scalably via GPs	[20]
MSSM/ Harvard	ACR	Acceptable Concentration Range model	New class of nonlinear statistical models for human data that incorporates and evaluates regulatory guideline values into analyses of health effects of exposure to chemical mixtures. Allows for human data to suggest points of departure for comparison to in vivo estimates from single chemicals.	[21]
MSSM/ Harvard	Mult DLAG	Multiple exposure distributed lag models with variable selection	A method to identify the presence of time-dependent interactions (interactions among chemical exposures experienced during different exposure windows) in a critical windows analysis. Identifies critical windows of exposure to multiple chemicals, and whether exposures experienced at different developmental windows interact with one another on a health outcome.	[22]
MSSM/ Harvard	BKMR-DLM	Bayesian Kernel Machine Regression-Distributed Lag Model	Develops distributed lag models for assessing critical windows of exposure associated with a mixture. The model simultaneously estimates a time-weighted combination of each exposure and estimates a multivariate exposures-response surface of these time-weighted exposures using BKMR.	[23]
MSSM/ Harvard	CVEK	Cross-validated kernel ensemble	Performs tests of interaction between two sets of exposures (i.e., two mixtures) while placing minimal assumptions on the main effects of each mixture. Asks whether one mixture (e.g., a collection of nutrients) modifies the effect of another (e.g., a metal mixture) as a whole.	[24]
MSSM/ Harvard	Bayes Tree Pairs	Bayesian Regression Tree Pairs	Estimates critical windows of susceptibility to an environmental mixture. Uses an additive ensemble of tree pairs to estimate main effects and interactions between time-resolved predictors with variable selection.	[25]
MSSM/ Harvard	DLMtree	Bayesian Treed Distributed Lab Models	Distributed lag linear and non-linear models. Method to improve the precision of critical window identification compared to methods that use spline or penalized spline basis functions. Interest focuses on identifying critical windows of exposure using data on a single exposure measured over time.	[26]
MSSM/ Harvard	Het-DLM	Heterogeneous distributed lag models	Methods for precision children’s environmental health—that is, methods to identify subject characteristics (child sex, maternal age, etc.) that modify distributed lag effects of exposure. Addresses which subjects exhibit the strongest associations with an exposure measured over multiple developmental windows, and whether the critical windows of exposure vary among subgroups.	[27]
MSSM/ Harvard	LWQS	Lagged Weighted Quantile Sum (WQS) regression	Uses a reverse distributed lag model for assessing critical windows of exposure associated with a mixture when the exposure temporal pattern differs across subjects. Can also incorporate strata-specific associations. Useful for identifying time-varying associations of a mixture effect and later life health/developmental outcomes.	[28]
MSSM/ Harvard	NLinteraction	Bayesian semiparametric regression with sparsity inducing priors	Estimates effects of environmental mixtures to allow for interactions of any order. Provides variable importance measures for both main effects and interactions among exposures within a mixture, while making minimal assumptions on the forms of those effects.	[29]
MSSM/ Harvard	RH-WQS	Repeated holdout Weighted Quantile Sum (WQS) regression	Generalizes WQS regression to include repeated holdout random data splits. Estimates a mixture effect using an empirically estimated weighted index.	[30]
MSSM/ Harvard	SGP-MPI	Scalable Gaussian Process regression via Median Posterior Inference	Takes a split-and-conquer strategy to fitting BKMR to big data. Yields summaries of the multivariate exposure-response surface, as well as variable importance measures of each individual exposure.	[31]
ND/Rice	BDS	Bayesian Data Synthesis	A Bayesian framework used to simulate fully synthetic datasets of mixed data types. The dataset may be comprised of mixed categorical, binary, count, and continuous datatypes. Can handle missing data and has customized metrics for attributing risk disclosure and other privacy concerns.	[32]
ND/Rice	BSSVI	Bayesian subset selection and variable importance for interpretable prediction and classification	Used to collect and summarize all near-optimal subset models to provide a complete predictive picture. Useful in the presence of correlated covariates, weak signals, and/or small sample sizes, where different subsets may be indistinguishable in their predictive accuracy.	[33]
ND/Rice	BVSM	Bayesian variable selection for understanding mixtures in environmental exposures	Variable selection via sparse summaries of a linear regression model. Given a Bayesian regression model with social and environmental covariates, addresses which variables matter most for predicting educational outcomes.	[34]
ND/Rice	FOTP	Fast, optimal, and targeted predictions using parameterized decision analysis	Computes targeted summaries and prediction for specific decision tasks. Given a target (or functional) of interest and a Bayesian model, constructs accurate, simple, and efficient predictions of future values or functionals of future values. Model summaries can be customized for each functionality.	[35]
ND/Rice	SCC	Spatiotemporal case-crossover	Presents a strategy for the case-crossover study design in a spatial-temporal setting. Incorporates a temporal case-crossover and a geometrically aware spatial random effect based on the Hausdorff distance.	[36]
ND/Rice	SiBAR	State Informed Background Removal	Computational technique to quantify ‘background’ versus ‘source influenced’ contributions to air pollutant time series. Addresses whether a hidden Markov model can be used and what the ‘background’ levels of pollutants are measured across an urban area.	[37]
UI Chicago	MVNimpute	Imputation of multivariate data by normal model	Implements multiple imputation to the data when there are missing and/or censored values.	[38]
UI Chicago	SPORM	Semi-Parametric Odds Ratio Model	Flexible semiparametric model for estimating complex relationship among multiple variables. Associations are modeled by odds ratio functions.	[14,39]
UI Chicago	TEV	Estimation and inference on the explained variation parameter	Estimates the explained variation of an outcome by a set of mixture pollutants.	[40,41]

^1^ Listed in alphabetical order, by institution. Project details available at NIH RePORTER: https://reporter.nih.gov/, accessed on 21 December 2021. Institutions: Columbia University Mailman School of Public Health, University of Illinois Chicago, Icahn School of Medicine at Mount Sinai, Harvard T.H. Chan School of Public Health, University of Notre Dame, Rice University, Boston University School of Public Health, Duke University.

**Table 3:** PRIME Methods by Research Question ^1^.

Method Acronym ^2^	Overall Effect Estimation	Toxic Agent Identification (Variable Selection)	Pattern Identification	A Priori Defined Groups	Interactions and Non-Linearities
FIN	X	X	X		X
BSSVI	X	X	X		X
SGP-MPI	X	X			X
RH-WQS	x	X			
Mult DLAG	X	X			X
MatchAlign	X	X			X
LWQS	x	X			
GriPS	X	X			X
DLMtree	X	X			X
DAG analysis	X	X			
BVSM	X	X			X
BMIM	X	X		X	X
BKMR-DLM	X	X			X
BKMR-CMA	X	X		X	X
Bayes Tree Pairs	X	X			X
ACR	X	X			
SPAMTREE	X		X		X
FOTP	X		X		X
BAG	X		X		X
TEV	X				X
SCC	X				
GL-GPs	X				X
BDS	X				X
SPORM		X	X	X	X
SiBAR		X	X		
BS3FA		X	X		
NLinteraction		X			X
Het-DLM		X			
BMC		X			
PFA			X		
PCP			X		
MrGap			X		
MixSelect			X		
GIF-SIS			X	X	
BN2MF			X		
CVEK				X	X

^1^ Research questions following Gibson et al., 2019 review: (1) Overall effect estimation: What is the overall effect of the mixture and what is the magnitude of association? (2) Toxic agent identification: Which congeners or chemicals are associated with the outcome? What congeners/chemicals are most important? (3) Pattern identification: Are there specific exposure patterns in the data? These can be managed with clustering and dimension reduction methods. (4) A priori defined groups: What are the associations between an outcome and a priori defined groups of exposures? (5) Interactions and non-linearities: Are there interactions between exposures? (6) Exposure-response relationship: What is the exposure-response relationship between each chemical and the outcome? Because almost all methods that investigate interactions also characterize potentially nonlinear exposure-response functions, we group questions #5 and #6 into a single column in this Table. ^2^ Method acronyms: ACR: Acceptable Concentration Range model; Bayes Tree Pairs: Bayesian Regression Tree Pairs; BAG: Bag of DAGs; BDS: Bayesian Data Synthesis; BKMR-CMA: Bayesian Kernel Machine Regression Causal Mediation Analysis; BKMR-DLM: Bayesian Kernel Machine Regression-Distributed Lag Model; BMC: Bayesian Matrix Completion for hypothesis testing; BMIM: Bayesian Multiple Index Model; BN2MF: Bayesian Non-parametric non-negative Matrix Factorization; BS3FA: Bayesian partially supervised sparse and smooth factor analysis; BSSVI: Bayesian subset selection and variable importance for interpretable prediction and classification; BVSM: Bayesian variable selection for understanding mixtures in environmental exposures; CVEK: Cross-validated kernel ensemble; DAG analysis: Directed Acyclic Graphs Analysis; DLMtree: Bayesian Treed Distributed Lab Models; FIN: Factor analysis for interactions; FOTP: Fast, optimal, and targeted predictions using parameterized decision analysis; GIF-SIS: General; zed infinite factor model; GL-GPs: Graph Laplacian based Gaussian Process; GriPS: Computational improvements for Bayesian multivariate regression models based on latent meshed Gaussian processes; Het-DLM: Heterogeneous distributed lag models; LWQS: Lagged Weighted Quantile Sum (WQS) regression; MatchAlign: Resolving rotational ambiguity in matrix sampling; MixSelect: Identifying main effects and interactions among exposures using Gaussian processes; MrGap: Manifold Reconstruction via Gaussian Process; Mult DLAG: Multiple exposure distributed lag models with variable selection; MVNimpute: Imputation of multivariate data by normal model; NLinteraction: Bayesian semiparametric regression with sparsity inducing priors; PCP: Principal Component Pursuit; PFA: Perturbed factor analysis; RH-WQS: Repeated holdout Weighted Quantile Sum (WQS) regression; SCC: Spatiotemporal case-crossover; SGP-MPI: Scalable Gaussian Process regression via Median Posterior Inference; SiBAR: State Informed Background Removal; SPAMTREE: Spatial Multivariate Trees; SPORM: Estimating complex relationship among outcome, biomarkers, and exposures; TEV: Estimation and inference on the explained variation parameter.

## Data Availability

Data and code from this work is available in the NIEHS PRIME GitHub: https://github.com/niehs-prime, accessed on on 16 November 2021, as well as individual investigator GitHub sites and/or publications (see Table S1).

## Supplementary Materials

The following supporting information can be downloaded at: https://www.mdpi.com/article/10.3390/ijerph19031378/s1, Table S1. PRIME Methods Details and Links to Software.
