# Supplementary material for: Powering Research through Innovative Methods for Mixtures in Epidemiology (PRIME) Program: Novel and Expanded Statistical Methods
Source: Int J Environ Res Public Health. 2022 Jan 26;19(3):1378. doi: 10.3390/ijerph19031378 (PMC8835015; doi:10.3390/ijerph19031378)
Supplement: Supplementary file 1 [file ijerph-19-01378-s001.zip › ijerph-1545258-supplementary.pdf]

Table S1. PRIME Methods Details and Links to Software

Project[1]	Method Acronym	Method Title	Use when…	Software[2]
**BU/ Harvard**	BKMR-CMA	Bayesian Kernel Machine Regression-Causal Mediation Analysis	Apply when interest focuses on causal mediation analysis, and data exists on an environmental mixture, a continuous mediator, and a continuous outcome.	https://github.com/kdevick/BKMR-CMA
**BU/ Harvard**	BMIM	Bayesian Multiple Index Model	Use for examining non-linear, non-additive relationships between exposures and outcome while reducing dimensionality via choice of indices.	https://github.com/glenmcgee/BMIM
**BU/ Harvard**	DAG analysis	Use of causal methods for determining which exposures to include in a model	Use when modeling causal relationships between multiple exposures and a health outcome.	No specific software
**Columbia**	BN2MF	Bayesian Non-parametric non-negative Matrix Factorization	Same as PCP	https://github.com/lizzyagibson/BN2MF
**Columbia**	PCP	Principal Component Pursuit	When the aim is to identify exposure patterns in the population (either common sources or behaviors); preferably somewhat high-dimensional exposures for the process to be meaningful	https://github.com/Columbia-PRIME/pcpr; https://github.com/Columbia-PRIME/PCPhelpers
**Duke**	BAG	Bag of DAGs	Modeling spatiotemporal data that may be large and have non-stationary or direction dependence; for example, due to the impact of winds on air pollution dispersal	https://github.com/jinbora0720/BAG
**Duke**	BMC	Bayesian Matrix Completion for hypothesis testing	1) large missingness in data having multiple chemicals and multiple assay endpoints, and/or 2) want to predict activity of a new (chemical, assay endpoint) pair, and/or 3) dose-response shapes are irregular, and/or 4) heteroscedastic signals are evident	https://github.com/jinbora0720/BMC
**Duke**	BS3FA	Bayesian partially supervised sparse and smooth factor analysis	1) data has functional Y and numeric (continuous, binary, count) data X.<br>2) no supervising X data are available and smooth unsupervised factor analysis is desired.<br>3) want to compute distance of chemicals relevant to toxicity<br>4) want to predict activity for a new chemical without dose-response information	https://github.com/niehs-prime/bs3fa
**Duke**	FIN	Factor analysis for interactions	1) predictors are correlated and<br>2) want to model pairwise/higher-order interactions of many predictors	https://github.com/niehs-prime/factor_interactions
**Duke**	GIF-SIS	Generalized infinite factor model	1) Want to fit a member of the large class of factorization/factor models<br>2) Variables have relationships which are known a priori and should inform shrinkage behavior	https://github.com/lorenzo-schiavon/GIF_SIS
**Duke**	GL-GPs	Graph Laplacian based Gaussian Process	Interested in performing nonparametric regression on a domain that is highly restricted or nonlinear	https://github.com/wunan3/Diffusion-based-Gaussian-Process
**Duke**	GriPS	Computational improvements for Bayesian multivariate regression models based on latent meshed gaussian processes	Addressing how to efficiently solve the big-n problem for GPs when the number of outcomes is large	https://cran.r-project.org/package=meshed
**Duke**	MatchAlign	Resolving rotational ambiguity in matrix sampling	1) post-processes MCMC samples to allow inference on unidentifiable random matrices (e.g., factor loadings matrix)	https://github.com/poworoznek/infinitefactor
**Duke**	MixSelect	Identifying main effects and interactions among exposures using Gaussian processes	1) Want to decompose an outcome into linear effects, interactions, and non-linear effects of the predictors; 2) have hierarchical variable selection on main effects and interactions.	https://github.com/fedfer/MixSelect

Project[1]	Method Acronym	Method Title	Use when…	Software[2]
**Duke**	MrGap	Manifold Reconstruction via Gaussian Process	1) Want to obtain an estimate of a low-dimensional manifold in the original high-dimensional observation space; 2) Data are noisy	https://github.com/wunan3/Manifold-reconstruction-via-Gaussian-processes
**Duke**	PFA	Perturbed factor analysis	A set of variables (e.g., chemical exposures) are expected to exhibit a similar covariance structure across multiple groups of observations (e.g., individuals from different demographic backgrounds) and the researcher would like to express that common structure via a set of shared factors.	https://github.com/royarkaprava/Perturbed-factor-model
**Duke**	SPAMTREE	Spatial Multivariate Trees	Multiple Gaussian outcomes, massive datasets (big n), some outcome measured at fewer input levels	https://cran.r-project.org/package=spamtree
**MSSM /Harvard**	NLinteraction	Bayesian semiparametric regression with sparsity inducing priors	Use this to estimate PODs from human data which may suggest data-driven uncertainty factors in risk assessments of single chemicals	https://github.com/jantonelli111/NLinteraction
**MSSM/ Harvard**	ACR	Acceptable Concentration Range model	Use when interest focuses on identifying critical windows of exposure, when data on multiple exposures measures with high temporal resolution (e.g., weekly during pregnancy) is available. Relative to BKMR-DLM, is better for large, administrative datasets.	https://github.com/danielmork/dlmtree
**MSSM/ Harvard**	Bayes Tree Pairs	Bayesian Regression Tree Pairs	Use when interest focuses on identifying critical windows of exposure, when data on multiple exposures measures with high temporal resolution (e.g., weekly during pregnancy) is available. Relative to BKMR-DLM, is better for large, administrative datasets. Relative to DLMtree, is better for modestly sized cohort studies.	https://github.com/niehs-prime/regimes
**MSSM/ Harvard**	BKMR-DLM	Bayesian Kernel Machine Regression-Distributed Lag Model	Use when interest focuses on sets of exposures, and how one set of exposures interacts with a set (e.g., Gene x Environment, Nutrition x Environment, Psychosocial Stress x Environment)	https://github.com/IrisTeng/CVEK-1
**MSSM/ Harvard**	CVEK	Cross-validated kernel ensemble	Apply when interest focuses on identifying a non-linear effect of an exposure and the critical window during which this exposure operates (e.g., specific weeks during pregnancy) on a health outcome measured in the future.	https://github.com/danielmork/dlmtree
**MSSM/ Harvard**	Het-DLM	Heterogeneous distributed lag models	Interest focuses on fitting distributed lag models to identify critical windows of a time-varying exposure, and on identifying subpopulations that are particularly vulnerable to this exposure (i.e exhibit stronger distributed lag effects).	https://github.com/danielmork/dlmtree
**MSSM/ Harvard**	LWQS	Lagged Weighted Quantile Sum (WQS) regression	Use when interest focuses on identifying critical windows of exposure and when the exposure temporal pattern differs across subjects	https://mran.microsoft.com/snapshot/2020-02-28/web/packages/gWQS/vignettes/gwqs-vignette.pdf; https://cran.r-project.org/web/packages/lwqs/vignettes/lwqs-vignette.html
**MSSM/ Harvard**	Mult DLAG	Multiple exposure distributed lag models with variable selection	Data includes measures of multiple exposures measured with high temporal resolution over a developmental window, and interest focusses on critical windows of exposure, and whether there are time-dependent interactions (does exposure of one pollutant early in time make an individual more susceptible to later exposures?)	https://github.com/jantonelli111/BayesianDLAG
**MSSM/ Harvard**	RH-WQS	Repeated holdout Weighted Quantile Sum (WQS) regression	Use with data on a continuous outcome and an environmental mixture, and interest primarily on interaction among exposures within a mixture, and the form of the multivariate exposure-response surface is potentially complex.	https://github.com/jantonelli111/Nlinteraction

Project[1]	Method Acronym	Method Title	Use when…	Software[2]
**MSSM/ Harvard**	SGP-MPI	Scalable Gaussian Process regression via Median Posterior Inference	Use when interest includes generalization of results by addressing variability of weights and association parameters.	https://github.com/evamtanner/Repeated_Holdout_WQS; https://cran.r-project.org/web/packages/gWQS/vignettes/gwqs-vignette.html
**MSSM/ Harvard**	DLMtree	Bayesian Treed Distributed Lab Models	Interest lies in applying BKMR to massive data.	https://github.com/danielmork/dlmtree
**ND/Rice**	BDS	Bayesian Data Synthesis	When a dataset cannot be shared publicly, but analyses on it are published. The dataset may be comprised of mixed categorical, binary, count and continuous datatypes	https://github.com/drkowal
**ND/Rice**	BSSVI	Bayesian subset selection and variable importance for interpretable prediction and classification	Use this when the goal is to identify which variables jointly matter for prediction	https://github.com/drkowal/BayesSubsets
**ND/Rice**	BVSM	Bayesian variable selection for understanding mixtures in environmental exposures	Use this when the goal is to select variables and provide uncertainty quantification for a linear model	https://github.com/drkowal/BayesSubsets
**ND/Rice**	FOTP	Fast, optimal, and targeted predictions using parameterized decision analysis	Use this when the inferential target(s) can be represented as a functional of the data and the goal is either to predict the target(s) or interpret the model via the target(s)	https://github.com/drkowal
**ND/Rice**	SCC	Spatiotemporal case-crossover	Use this when information is available for spatial regions (e.g., census blocks) over time and when the constant exposure assumption is not reasonable and spatial regions are irregular.	https://github.com/kathyensor
**ND/Rice**	SiBAR	State Informed Background Removal	Interested in apportioning pollutants to local sources	https://github.com/bactkinson/SIBaR_Background_Removal_and_Quantification
**UI Chicago**	MVNimpute	Imputation of multivariate data by normal model	Preparing data for analysis	https://github.com/niehs-prime/mvnimpute
**UI Chicago**	SPORM	Semi-Parametric Odds Ratio Model	Relationship among exposures, biologic intermediates and health outcomes are of major interest	https://github.com/hychen-uic/SPORM
**UI Chicago**	TEV	Estimation and inference on the explained variation parameter	Exposures are of high-dimensional and the effects of the exposures are weak and dense.	https://github.com/hychen-uic

[1] Listed in alphabetical order, by institution. Project details available at NIH RePORTER: https://reporter.nih.gov/. Institutions: Columbia University Health Sciences, University of Illinois at Chicago, Icahn School of Medicine at Mount Sinai, Harvard University T.H. Chan School of Public Health, University of Notre Dame, Rice University, Boston University Medical Campus, Duke University.
[2] Updates to the software are expected over time, including links to packages not yet deposited. To retrieve the most up to date software, query GitHub https://github.com/ using the method title or acronym.
